# Imaging the premonitory phase of migraine - new insights into generation of the migraine attack

**DOI:** 10.1186/1129-2377-14-S1-P112

**Published:** 2013-02-21

**Authors:** FH Maniyar, T Sprenger, C Schankin, PJ Goadsby

**Affiliations:** 1 Pinderfields General Hospital, NHS Trust, UK; 2 University Hospital Basel, Switzerland; 3 University of California, San Francisco, USA

## Background and introduction

When questioned closely the majority of migraineurs report premonitory symptoms before headache that represent the earliest clinical manifestations of the attack [1]. The premonitory phase has not been hitherto imaged and offers an opportunity to understand fundamental aspects of the disorder.

## Objective

To image brain areas involved in the premonitory phase of migraine.

## Methods

We included patients with episodic migraine without aura, not taking preventive medications that experienced premonitory symptoms before headache. Patients were triggered with intravenous nitroglycerin on a first occasion to select those who responded with habitual premonitory symptoms and delayed headache which resembled their migraine [2]. On a second occasion, the triggering was repeated and PET scans were performed with H215O during baseline, premonitory phase(no pain) and delayed headache. The main outcome was comparing the first premonitory scans of all patients versus the baseline scans of all patients (n=8) using statistical parametric mapping [3].

## Results

Tiredness, neck stiffness and increased thirst were the three most common premonitory symptoms during the scanning session. All patients had either right-sided or bilateral with predominantly right-sided delayed headache. Comparing the first premonitory scans of all patients versus baseline scans of all patients, we found activations in the postero-lateral hypothalamic region, adjacent midbrain ventral tegmental area in the region of substantia nigra, peri-aqueductal grey and dorsal pons in the region of locus coeruleus. We also found activations in bilateral occipital cortex, right temporal cortex and bilateral but predominantly right-sided prefrontal cortex.

## Conclusion

Hypothalamic and brainstem structures are activated early in migraine - during the premonitory phase, before the appearance of headache. Hypothalamic involvement can explain many of the premonitory symptoms and also the reason why change in homeostasis triggers migraine. This study was funded by a grant from the Sandler Family Trust.
